# Bodily synchronization and ecological validity: a relevant concern for nonlinear dynamical systems theory

**DOI:** 10.3389/fnhum.2015.00064

**Published:** 2015-02-13

**Authors:** Roberto Musa, David Carré, Carlos Cornejo

**Affiliations:** ^1^Laboratorio de Lenguaje, Interacción y Fenomenología, Escuela de Psicología, Pontificia Universidad Católica de ChileSantiago, Chile; ^2^Centre for Cultural Psychology, Aalborg UniversityAalborg, Denmark

**Keywords:** synchronization, nonlinear dynamical systems, joke, interpersonal coordination, movement

In their recent article “Bodily synchronization underlying joke telling,” Schmidt et al. (2014) argue that two-person neuroscience is an insufficient approach to explain interpersonal coordination. Interpersonal entrainment, they claim, must be understood bearing in mind that the whole person is embedded in an embodied and social situation. To this effect, they present novel motion-capture data on bodily coordination during a knock-knock joke telling task. Schmidt et al. (2014) make a valid point in calling attention to the complexity of synchronization activity. Precisely because of its importance, however, we believe it is necessary to highlight methodological and substantive caveats that render their work ultimately unsuccessful in accounting for human coordination in natural social interactions.

Schmidt et al. (2014) characterize their research perspective—behavioral dynamics—as one that “uses concepts and tools from nonlinear dynamical systems to tackle social entrainment as an instance of self-organization, where individuals form a social unit, a dynamical interpersonal synergy, *without planning*, to *construct meaningful actions together*” (p. 2, emphases added). Yet when their experimental procedure is examined, little of the interaction between the test subjects can be considered meaningful or unplanned. Prior to the experiment, participants were asked to engage in ice-breaking tasks, so that they “became accustomed to coordinating their movements” (Schmidt et al., 2014, p. 4). Also, by the time the experiment began, participants were already habituated to the jokes they would have to tell—and listen to—having been asked to “familiarize themselves with the joke's lines and their concomitant puns” (Schmidt et al., 2014, p. 4). Any element of surprise at the joke's punch line was therefore lost. We are informed furthermore that the entire series—which consisted of ten jokes—was read aloud six times throughout the experiment. Such a high degree of repetition could compromise participants' understanding of the meaning of the jokes, producing semantic satiation (Falk et al., 2014). Thus, the procedure that subjects are told to engage in can hardly be considered a “joke telling ‘dance”’ (Schmidt et al., 2014, p. 3), unless: (a) we understand “dance” as a pre-established, repeatedly trained sequence of steps; and (b) we think that repeating a joke script six times has no impact on the sense of a natural joke telling interaction, namely being funny. The fact that no data is reported regarding how enjoyable participants found the jokes, even though said reports were collected, is telling.

A further threat to ecological validity is the fact that the temporal structure of the joke telling ritual did not emerge naturally during the interaction. It was rather imposed by the experimenters who “demonstrated the pace of telling so that each joke takes approximately 5–7 s” (Schmidt et al., 2014, p. 5) in such a manner that “each of the first four lines is said isochronously, 1 beat each for each line, comprising 4 beats for the setup of the joke; and typically the last (punch) line is given 4 beats (2 beats to say the line and 2 beats pause before the next joke) for the conclusion of the joke” (Schmidt et al., 2014, p. 4). This procrustean timetable must be understood as in the benefit of the analyses, but we must not lose sight that it comes at considerable cost to what little naturalness remains of what should be, supposedly, a natural interaction. Hence, while Schmidt et al.'s (2014) intent of studying spontaneous, meaningful human activity is praiseworthy, the way in which the experiment was run is eerily reminiscent of the studies of artificial bodily movements from which they allegedly were trying to separate themselves.

Modifying a joke telling situation to the point of making it a stereotypical ritual is not irrelevant for behavioral entrainment insofar as human participants are not telling jokes anymore, but just following the experimenters' instructions verbatim (Cutica, 2007). Although physical in nature, bodily coordination is more than mere physical movement; rather, it is tightly related to communicative (Kendon, 1970) and social (Semin and Cacioppo, 2008) dimensions of human experience. Therefore, behavioral entrainment does not emerge as a by-product of human physical co-presence and joint actions, independently from the meaning of those actions. Hence, Schmidt et al. (2014) would have greatly enriched their outlook by taking the first-person perspective of their participants into account, as has been subtly done in recent studies (Froese et al., 2014a,b).

In a crucial point, Schmidt et al. (2014) argue that:

[B]ehavioral entrainment in social interactions can be understood using same self-organizing processes used to understand the entrainment of *mechanical oscillators* (e.g., pendulum clocks). In this way, social coordination is cast as an instance of synchronization phenomena found generically in nature, and thereby, *it can be measured and understood* using mathematical models of synchronization (p. 2, emphases added).

As seen, Schmidt et al.'s (2014) hinting that human interactions behave exactly like other coupled mechanical oscillators understates the importance of communicative and social facets of bodily coordination. In order to truly take these dimensions into account, nonlinear dynamical systems theory must strive for designs that capture interaction in a more ecologically valid way, following their integrative aim (Dale et al., 2013). In this case, the unnatural way in which participants were asked to perform had little to do with those everyday social interactions we call joke telling. Designing experiments that more appropriately capture the richness and complexity of real human interaction is crucial, but we must also ask ourselves what we can learn about human coordination by knowing that the movements of interacting persons can be modeled similarly to pendulum clocks, hurricane displacements or neural activity in the olfactory bulb. Describing a Van Gogh self-portrait as an arrangement of oil particles may be certainly right; but in doing so we are missing the most important level of description for such an object. In the same way, human coordination involves regular physical movements indeed, but its interactional meaning is what defines it as human coordination, and not a mere physical co-occurrence. It is precisely this meaning that is missing from Schmidt et al.'s account.

## Conflict of interest statement

The authors declare that the research was conducted in the absence of any commercial or financial relationships that could be construed as a potential conflict of interest.
